# qHBsAg for the Identification of Liver Histological Abnormalities in HBeAg-Negative Chronic Hepatitis B Patients with Normal and Mildly Elevated ALT Levels

**DOI:** 10.1155/2022/8695196

**Published:** 2022-07-14

**Authors:** Qinyi Gan, Yan Huang, Chuanwu Zhu, Shuang Zhao, Haoshuang Fu, Minghao Cai, Jiexiao Wang, Chenxi Zhang, Simin Guo, Zhujun Cao, Qing Xie

**Affiliations:** ^1^Department of Infectious Diseases, Ruijin Hospital, Shanghai Jiao Tong University School of Medicine, Shanghai 200025, China; ^2^Department of Infectious Diseases, The Fifth People's Hospital of Suzhou, Suzhou 215131, China

## Abstract

**Backgrounds:**

Noninvasive detection of histological abnormalities remains challenging in patients with HBeAg-negative chronic HBV infection with normal or mildly elevated levels of alanine aminotransferase (ALT). This study aimed to assess the utility of serum quantitative hepatitis B surface antigen (qHBsAg) in identifying significant histological lesions in this population.

**Methods:**

This is a single-center study with retrospective analysis of 392 treatment-naive patients of HBeAg-negative chronic HBV infection with normal or mildly elevated levels of ALT.

**Results:**

In this cohort, significant necroinflammation and fibrosis were found in 69.4% and 61.5% of patients, respectively. Patients with qHBsAg >1000 IU/mL (*N* = 236) had more hepatic inflammation of ≥*G*2 (75.4% vs. 60.9%, *P* < 0.01) or fibrosis ≥ *S*2 (66.1% vs. 54.5%, *P* < 0.05) compared to those without (*N* = 156). Serum HBsAg (cutoff point = 1000 IU/mL), aspartate aminotransferase (AST) level (cutoff point = 25 IU/L), age (cutoff point = 40 years), and HBV family history were identified as independent predictors of significant histological abnormalities in multivariate logistic analysis.

**Conclusions:**

A significantly higher proportion of patients with histological abnormalities were found in patients with qHBsAg >1000 IU/mL than those without. The qHBsAg level together with age, AST, and family history of HBV infection could be used as an algorithm to help noninvasive patient selection for antiviral therapy.

## 1. Introduction

Chronic hepatitis B virus (HBV) infection is one of the common causes of chronic liver diseases and remains a global public health threat. During the natural course of HBV infection, around 15–40% will develop HBV-related complications including hepatitis B flare-up, liver cirrhosis, and hepatocellular carcinoma (HCC) [1]. Patients with evidence of ongoing hepatic inflammation or fibrosis are recommended for antiviral therapies that can significantly reduce the risk of HBV-related complications. However, it remains controversial in the antiviral treatment of patients with normal or mildly elevated levels of alanine aminotransferase (ALT), particularly in those with HBeAg-negative infection. In such indeterminate cases, a histological assessment of specimens through liver biopsy is recommended by the current guidelines, whereas the application of this procedure is largely limited by its invasive nature [2–4]. Noninvasive alternatives to liver biopsy for the assessment of disease severity in patients with HBeAg-negative infection are needed.

In untreated patients, quantitative hepatitis B surface antigen (qHBsAg) declines slowly through the natural course and remains stable for a long time after HBeAg seroconversion. Several studies reported that a qHBsAg level below 1000 IU/ml in HBeAg-negative patients was strongly associated with the inactive carrier state, especially in patients with a low serum level of HBV DNA and normal ALT [5–7]. Thus, we used this cutoff value to classify patients with low and high qHBsAg levels and explored the relationship between qHBsAg and liver histological changes.

The current study is designed to assess the utility of qHBsAg in distinguishing significant histological abnormalities in treatment-naive HBeAg-negative chronic HBV infection patients with normal or mildly elevated ALT levels.

## 2. Materials and Methods

### 2.1. Patients

This is a single-center, cross-sectional observational study of HBeAg-negative treatment-naive CHB patients with normal or mildly elevated ALT levels. All patients underwent liver biopsy for the assessment of a liver disease between January 2009 and December 2019. Inclusion criteria were defined as follows: (i) aged older than 18 years; (ii) with positive serum hepatitis B surface antigen for at least 6 months; and (iii) with serum HBeAg negative. Exclusion criteria were defined as follows: (i) with HCC or other chronic liver diseases such as other viral hepatitis, autoimmune hepatitis, and nonalcoholic fatty liver disease; (ii) with previous history of any antiviral therapy; (iii) with unqualified liver samples, defined as less than 10 mm or containing less than six portal triads; and (iv) with ALT >80 IU/L. This study complied with the Declaration of Helsinki and was approved by the Institutional Ethics Review Committee at Ruijin hospital.

### 2.2. Clinical and Histological Evaluation

Demographical, laboratory, and histological data were collected within one month before liver biopsy. The upper limit of normal (ULN) for ALT was 40 U/L according to the EASL criterion. Qualitative HBsAg and HBeAg analyses were performed by AxSYM or Architect assays (Abbott, Abbott Park, IL, USA). All patients provided written informed consent for liver biopsy. The indications for liver biopsies are as follows: (1) staging of inflammation and fibrosis to guide antiviral therapy and (2) differential diagnosis of other liver diseases other than HBV infection. Histologic grading of necroinflammation (G0–G4) and staging of liver fibrosis (S0–S4) were performed according to the Scheuer scoring system by two senior liver pathologists blinded to clinical data/biological data. Patients with *G* ≥ 2 (significant inflammation) and/or *S* ≥ 2 (significant fibrosis) were considered as having significant histological abnormalities. These patients are indicated for antiviral therapy irrespective of the ALT level according to the AASLD [4], EASL [3], and APASL [2] practice guidelines.

### 2.3. Statistical Analysis

Categorical variables were described as count (percentages). Continuous variables with normal distribution were presented as mean ± standard deviation (SD), otherwise as median (interquartile range, IQR). Means for continuous variables were compared using independent group *t*-tests when the data were normally distributed; otherwise, the Mann–Whitney test was used. Categorical variables were compared using the *χ*^2^ test or Fisher's exact test as appropriate. Univariate logistic regression was performed to identify the risk factors of significant histological abnormalities followed by multivariate logistic regression to adjust potential confounding effects. The optimum cutoff values for identification of significant histological changes were selected by maximizing the sum of sensitivity and specificity. All statistical analyses were performed using GraphPad Prism 7.0 (GraphPad Software, San Diego, California, USA) or SPSS 24.0 (IBMCorp., Armonk, New York, USA). A two-tailed *P* value of <0.05 was considered statistically significant.

## 3. Results and Discussion

### 3.1. Baseline Characteristics of Patients

**Figure 1:**
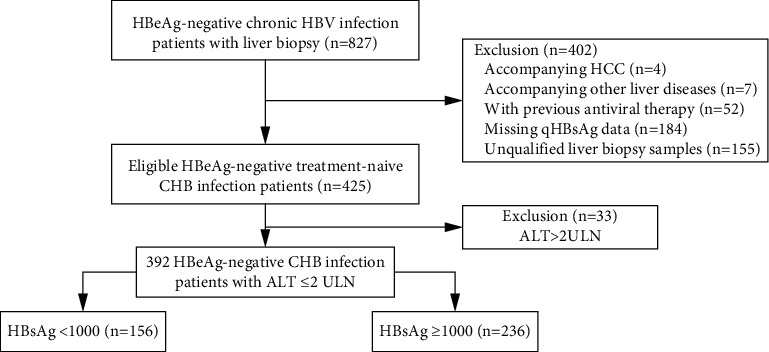
Patient flow diagram.

A total of 827 patients with HBeAg-negative chronic HBV infection were screened and liver biopsy results were reviewed and 392 of them were eligible for analysis (Figure 1). Patients were divided into high (qHBsAg ≥1000 IU/mL, *N* = 236, 60.2%) and low HBsAg groups (qHBsAg <1000 IU/mL, *N* = 156, 39.8%). Baseline characteristics are shown in Table 1. The median age was 42 years and 249 (63.5%) patients were male. ALT levels were normal in 272 (69.4%) patients. The median log10 HBsAg level of the overall cohort was 3.2 (2.6–3.5) IU/mL.

Patients in the high HBsAg group were significantly younger (*P*=0.021) with higher ALT (*P*=0.005), AST (*P*=0.027), GGT (*P*=0.001), and HBV DNA (*P*=0.004) levels compared to those in the low HBsAg group. Besides, the proportion of significant liver histological abnormality in the high HBsAg group was significantly higher than that in the low HBsAg group (84.3% vs. 67.9%, *P*=0.0001).

### 3.2. Relationship between the HBsAg Level and Liver Histology

**Figure 2:**
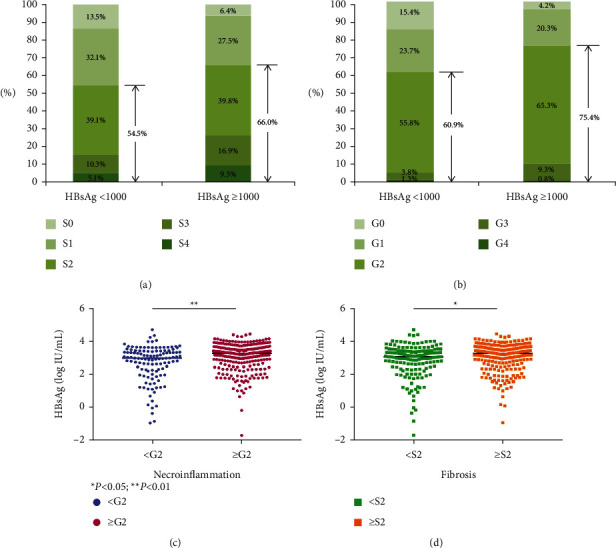
Relationship of HBsAg with liver necroinflammation and fibrosis.

Significant necroinflammation (75.4% vs. 60.9%, *P*=0.002) or fibrosis (66.0% vs. 54.5%, *P*=0.021) was more frequently observed in patients with high HBsAg than in those with low HBsAg, respectively (Figures 2(a) and 2(b)). The qHBsAg level was significantly higher than that in patients with G2–4 necroinflammation than that in those with G0-1 (3.1 vs. 2.7, *P*=0.0004, Figures 2(c) and 2(d)). Similarly, the qHBsAg level was significantly higher in patients with significant fibrosis than in those without (3.0 vs. 2.8, *P*=0.013).

### 3.3. Risk Factors Associated with Significant Histological Abnormalities

The HBsAg level (OR = 1.493, *P*=0.002), AST (OR = 1.057, *P*=0.001), and HBV family history (OR = 1.877, *P*=0.017) were identified as independent predictors for significant liver histopathology under multiple logistic regression analysis with stepwise selection (Table 2). Using the same analyses, the HBsAg level (OR = 1.634, *P*=0.0001), AST (OR = 1.048, *P*=0.002), or age (OR = 1.027, *P*=0.031) was identified as an independent predictor for significant necroinflammation and HBsAg level (OR = 1.299, *P*=0.026), AST (OR = 1.039, *P*=0.002), or HBV family history (OR = 1.725, *P*=0.013) for significant fibrosis (Table 2). The optimal cut points of AST, age, and qHBsAg for identification of significant histological changes were 24.5, 42.5, or 3.1, respectively.

### 3.4. Probability of Histological Abnormalities Based on the HBsAg Level, Age, and AST

**Figure 3:**
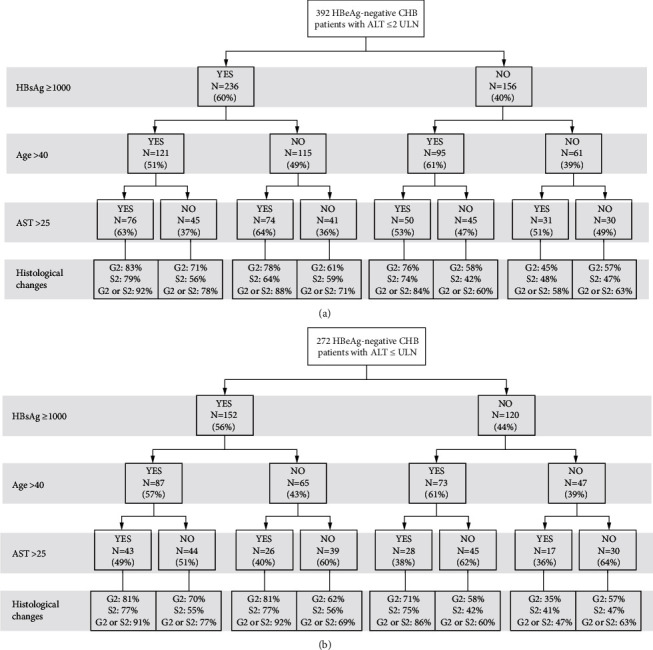
Chances of histological abnormalities among HBeAg-negative chronic HBV infection patients based on HBsAg level , AST level, and age.

According to the statistical cutoff values by ROC curves and values recommended in the guidelines, we calculated percentages of significant histological abnormalities in our cohort based on HBsAg (higher or lower than 1000 IU/mL), AST level (higher or lower than 25 IU/L), and age (higher or lower than 40 years) as shown in Figure 3.

Among patients with ALT ≤2ULN, there were 76 (19.4%) patients with HBsAg ≥1000 IU/mL, age >40 years, and AST >25 IU/L. The proportion of patients with necroinflammation ≥ *G*2, fibrosis ≥ *S*2, and necroinflammation ≥ *G*2 or fibrosis ≥ *S*2 were 83%, 79%, and 92%, respectively. 30 (7.7%) patients had HBsAg ≤1000 IU/mL, age <40 years, and AST <25 IU/L. The proportion of patients with necroinflammation ≥ *G*2, fibrosis ≥ *S*2, and necroinflammation ≥ *G*2 or fibrosis ≥ *S*2 were 57%, 47%, or 63%, respectively.

Among patients with ALT ≤ ULN, there were 43 (15.8%) patients with HBsAg ≥1000 IU/mL, age >40 years, and AST >25 IU/L. The proportion of patients with necroinflammation ≥ *G*2, fibrosis ≥ *S*2, and necroinflammation ≥ *G*2 or fibrosis ≥ *S*2 were 81%, 77%, and 91%, respectively. 30 (7.7%) patients had HBsAg ≤1000 IU/mL, age <40 years, and AST <25 IU/L. The proportion of patients with necroinflammation ≥ *G*2, fibrosis ≥ *S*2, and necroinflammation ≥ *G*2 or fibrosis ≥ *S*2 were 57%, 47%, and 63%, respectively.

Predictive values for serum levels of HBsAg, AST, and age for identifying significant histological changes are shown in Table 3. A HBsAg level above 1000 IU/ml, AST level above 25 IU/L, and age above 40 years identified patients with significant histological changes with a positive predictive value (PPV) of 92.1% and specificity of 93.1%.

## 4. Discussion

qHBsAg has been proposed as a new diagnostic tool for the characterization of the HBV disease state. Recent reports suggest that HBsAg quantification might be a useful complement to HBV DNA quantification for clinical assessment and treatment monitoring in chronic HBV infection patients [7–9]. This study shows that qHBsAg is associated with liver inflammation and fibrosis assessed by histological scoring in treatment-naive HBeAg-negative chronic HBV infection patients with normal or mildly elevated ALT levels. Thus, such results can help clinicians to identify patients having significant liver necroinflammation or fibrosis who are in need of antiviral treatment.

Our study found that 77.8% treatment-naive HBeAg-negative chronic HBV infection patients with ALT ≤2ULN had a significant liver histological change. It is worth noting that 74.3% of patients with ALT ≤ ULN had a significant liver histological change, which is the criterion for starting an antiviral treatment. Therefore, it is critical to distinguish these patients using virological or biochemical markers without liver biopsy, so as to improve their clinical outcomes. Also, the proportion of patients with significant liver fibrosis and inflammation in the high HBsAg group was significantly higher than the low HBsAg group. Moreover, the high HBsAg group had higher ALT, AST, and GGT levels than the low HBsAg group, indicating that the HBsAg level can reflect the liver necroinflammatory activity. This is consistent with previous studies showing that the HBsAg level reflects a clinical stage and liver disease severity [6, 9]. However, there were also studies showing different results. One research reported that HBsAg levels were lower in more advanced liver fibrosis in HBeAg-negative CHB patients [10]. Another study showed HBsAg levels do not distinguish patients with significant fibrosis in HBeAg-negative chronic hepatitis B patients [11]. This could be explained by differences in HBV genotypes, the origin of HBsAg proteins, or phases of HBV infection.

As recommended by the AASLD guidelines, HBeAg-negative patients with ALT ≤2ULN are recommended to monitor the levels of ALT and HBV DNA or go through liver biopsy, instead of starting the antiviral treatment immediately [4]. However, studies have shown that these patients could also have significant liver histological changes, which require antiviral treatment [12–14]. In the present study, we further identified the HBsAg level, age, AST level, and HBV family history as independent risk factors for significant histological abnormalities. This is consistent with previously reported data that the AST level can reflect the activity of liver necroinflammation in HBeAg-positive CHB patients [15]. A previous study showed increasing age as an independent predictor of significant liver fibrosis in HBeAg-negative CHB patients with persistently normal ALT [16]. Also, patients with age >40 years were associated with a higher chance of significant histological disease according to the AASLD guideline [4].

The chances of histological abnormalities in our cohort indicated that the overall diagnostic accuracy for identifying significant liver histological abnormalities by the HBsAg level was better than the combination with HBV DNA or HBV DNA alone. Using HBsAg, AST, and age as parameters to identify patients with significant histological abnormalities showed a PPV of 92.1% and specificity of 93.1%, indicating it as an effective way for screening patients in need of antiviral treatment. According to our results, whether ALT ≤2ULN or ALT ≤ ULN, patients with HBsAg ≥1000 IU/mL, age >40 years, and AST >25 IU/L can start antiviral treatment without having biopsy, as about 90% of them had significant liver histological abnormalities. Patients who meet any two of the above criteria should assess disease severity with noninvasive tests or liver biopsy. Patients with HBsAg <1000 IU/mL, age <40 years, and AST <25 IU/L have a lower chance of histological abnormalities but still need close monitor of serum markers and perform a liver biopsy when necessary.

The current study has some limitations. As a retrospective cross-sectional study, the analysis was based on data from a single time test. The relatively higher percentage of patients with significant liver histological changes compared to other studies could be related to this reason. Also, data such as HBV genotype were missing because this test was not performed routinely in clinical practice. Although using HBsAg and AST levels and age as parameters to identify patients with significant histological changes showed high specificity and PPV, the sensibility and NPV are relatively low. Prospective studies in the future are still needed.

## 5. Conclusions

In conclusion, qHBsAg level helps to identify histological significant abnormalities in treatment-naive HBeAg-negative chronic HBV infection patients with normal or mildly elevated levels of ALT. An algorithm based on the qHBsAg, AST, age, and HBV family history further improves discriminative accuracy and could be a valuable tool to guide antiviral treatment.

## 6. Disclosure

QG, YH, and CZ share co-first authorship.

## Figures and Tables

**Table 1:** Baseline characteristics of HBeAg-negative chronic HBV infection patients based on HBsAg levels.

	All (*n* = 392)	HBsAg <1000 IU/mL (*n* = 156)	HBsAg ≥1000 IU/mL (*n* = 236)	*P* value
Age, years	42 (36–51)	44 (37–53)	41 (35–49)	0.0214
Male, *n* (%)	249 (63.5%)	99 (63.5%)	150 (63.6%)	0.9843
BMI	23.4 (21.5–25.6)	23.4 (21.7–25.2)	23.2 (21.5–26.1)	0.6318
HBV family history, *n* (%)	196 (50%)	73 (46.8%)	123 (52.1%)	0.3021
HCC family history, *n* (%)	58 (14.8%)	24 (15.4%)	34 (14.4%)	0.7896
Leukocyte count (×10^9^/L)	5.4 (4.6–6.4)	5.3 (4.7–6.2)	5.5 (4.6–6.4)	0.3255
Platelet count (×10^9^/L)	177 (148–209)	179 (148–211)	174 (146–207)	0.3151
ALT, IU/L	31 (22–43)	28 (21–40)	32 (23–44)	0.0051
ALT ≤ ULN, *n* (%)	272 (69.4%)	120 (76.9%)	152 (64.4%)	0.0085
AST, IU/L	27.5 (22.3–34)	26 (22–32)	28 (23–34)	0.0267
ALP, IU/L	70 (57–82) (*n* = 381)	69 (56–82) (*n* = 152)	70 (58–84) (*n* = 229)	0.2048
GGT, IU/L	20 (15–32) (*n* = 379)	18 (14–26) (*n* = 152)	23 (16–36) (*n* = 227)	0.0014
TB, µmol/L	14.2 (11.4–18.6)	14.7 (11.5–19.1)	14.1 (11.1–18.5)	0.3947
HBV DNA, log100 IU/mL	3.6 (2.9–4.5)	3.4 (2.7–4.1)	3.8 (3–4.9)	0.0037
HBsAg, log10 IU/mL	3.2 (2.6–3.5)	2.3 (1.7–2.7)	3.4 (3.3–3.7)	<0.0001
Necroinflammation ≥ G2, *n* (%)	273 (69.4%)	95 (60.9%)	178 (75.4%)	0.0022
Fibrosis ≥ S2, *n* (%)	241 (61.5%)	85 (54.5%)	156 (66.1%)	0.0207
Necroinflammation ≥ G2 or fibrosis ≥ S2, *n* (%)	305 (77.8%)	106 (67.9%)	199 (84.3%)	0.0001

ALT, alanine aminotransferase; AST, aspartate transaminase; ALP, alkaline phosphatase; GGT, gamma-glutamyltransferase; TB, total bilirubin abnormal.

**Table 2:** Multivariate analysis of clinical parameters independently associated with significant histological abnormalities.

Parameter	Necroinflammation ≥ *G*2			Fibrosis ≥ S2			Necroinflammation ≥ G2 or fibrosis ≥ *S*2		
	OR	95% CI	*P* value	OR	95% CI	*P* value	OR	95% CI	*P* value
HBsAg	1.634	1.274–2.097	<0.0001	1.299	1.031–1.637	0.026	1.493	1.157–1.927	0.002
AST	1.048	1.018–1.079	0.002	1.039	1.014–1.065	0.002	1.057	1.023–1.093	0.001
Age	1.027	1.002–1.053	0.031						
HBV family history				1.725	1.123–2.650	0.013	1.877	1.117–3.153	0.017

AST, aspartate transaminase.

**Table 3:** Predictive values for serum levels of HBsAg, AST, and age for identifying significant histological changes in HBeAg-negative patients (*n* = 392).

		*G*2 or *S*2 (ALT ≤80 IU/L)					
		Yes	NO	PPV (%)	Sensitivity (%)	NPV (%)	Specificity (%)
HBsAg ≥1000 IU/ml	YES	199	37	84.3	65.3	32.1	57.5
	NO	106	50				
Age >40 years	YES	174	42	80.6	57.1	25.6	51.7
	NO	131	45				
AST >25 IU/L	YES	195	36	84.4	63.9	31.7	58.6
	NO	110	51				
HBsAg ≥1000 IU/ml and age >40 years and ALT >25 IU/L	YES	70	6	92.1	23.0	25.6	93.1
	NO	235	81				

PPV, positive predictive value; NPV, negative predictive value.

## Data Availability

The datasets generated and analyzed during the present study are available from the corresponding author upon reasonable request.
